# Retraction Note: Cabozantinib induces PUMA-dependent apoptosis in colon cancer cells via AKT/GSK-3β/NF-κB signaling pathway

**DOI:** 10.1038/s41417-022-00545-3

**Published:** 2022-10-14

**Authors:** Shida Yang, Xiaobing Zhang, Huiling Qu, Bo Qu, Xiaoxue Yin, Hongmei Zhao

**Affiliations:** 1 Department of Laboratory Medicine, The people’s Hospital of Liaoning Province, Shenyang, China; 2 Department of Neurology, The people’s Hospital of Liaoning Province, Shenyang, China

Retraction to: *Cancer Gene Therapy* 10.1038/s41417-019-0098-6, published online 11 June 2019

The Editor-in-Chief has retracted this article at the authors’ request. After publication, the authors became aware that some images were misused in Fig. 6d, which resulted in image overlap between the *PUMA-*KD Control and Cabozantinib groups. The raw data are no longer available. Therefore, the authors have lost confidence in the conclusions drawn in the study.

Shida Yang, Xiaobing Zhang, Huiling Qu, Bo Qu and Xiaoxue Yin agree to this retraction. Hongmei Zhao has not responded to any correspondence from the editor or publisher about this retraction.

